# Advanced Age Worsens Phenotypes of Ocular Hypertension in Mice

**DOI:** 10.14336/AD.2025.0349

**Published:** 2026-05-28

**Authors:** Priyamvada M. Pitale, Solomon E. Gibson, Caroline C. Keehn, Arman T. Yazdian, Guofu Shen, Benjamin J. Frankfort

**Affiliations:** ^1^Department of Ophthalmology, Baylor College of Medicine, Houston, TX, USA.; ^2^Department of Neuroscience, Baylor College of Medicine, Houston, TX, USA.

**Keywords:** Aging, Retinal Vasculature, Ocular Hypertension, Glaucoma, Basement Membrane, Tight Junction

## Abstract

Glaucoma is a neurodegenerative disorder of the optic nerve and retinal ganglion cells (RGCs) and a major cause of blindness. The two most important risk factors for glaucoma are ocular hypertension (OHT) and advanced age. In this study, we explored the combined impact of aging and OHT on retinal neuronal and microvasculature health. We induced OHT using the bead-injection model in 12 week old (young) and 1.5 year old (old) mice and monitored intraocular pressure (IOP) for 2 weeks. We then explored vascular phenotypes, blood retinal barrier components, RGC counts, and electroretinogram (ERG) changes. Aged mice displayed reduced retinal microvasculature complexity, retinal vascular phenotypes in all three retinal capillary plexi (RCPs), and abnormal ERGs. Aging also impacted basement membrane (BM) and tight junction (TJ) morphology. The impact of OHT was much more evident in old mice; RGC loss was exacerbated, retinal vascular phenotypes were magnified across all three RCPs, and BM and TJ phenotypes were much more severe. However, the impact of OHT on retinal function was unchanged in old mice. Interestingly, the nature of these phenotypes was not equivalent among all RCPs, suggesting regional shared and distinct susceptibilities to aging and OHT. Taken together, aging causes multiple neurovascular phenotypes in mouse retinas, and OHT causes more severe effects in old mice. This suggests an interaction between aging and OHT that may help explain the increased prevalence of glaucoma in older humans.

## INTRODUCTION

Glaucoma, a neurodegenerative disease of retinal ganglion cells (RGCs) and the optic nerve, is a major cause of irreversible blindness globally [1-5]. Intraocular pressure (IOP) is the only modifiable glaucoma risk factor [6, 7] and age is the strongest non-modifiable risk factor [8, 9]. Age is also a major risk factor in many other neurodegenerative diseases such as Alzheimer’s and Parkinson’s diseases [8, 10, 11]. These disorders share similar epidemiologic patterns, including increased incidence beginning in mid- to late-adulthood, and increasing prevalence with increasing age [5, 9, 12, 13].

The mechanism by which age-related changes drive neurodegeneration is not well understood. In the central nervous system (CNS), damage to the neurovascular unit (NVU), composed of glial cells, microglial cells, neurons, pericytes, and endothelial cells, plays a critical role in the pathophysiology of neurodegeneration [14]. The intercommunication between neurons and their supporting capillary plexus regulates local blood flow and provides selective communication with the systemic circulation through the blood-brain barrier (BBB) and blood-retinal barrier (BRB) [14-17]. Studies of aging in CNS models have shown that inadequate ATP generation, altered metabolism of astrocytes and neurons, an increase in neuroinflammation, and a decrease in angiogenesis lead to NVU insufficiency [18-22]. In the retina, it has been reported that some of the key functions of the NVU, namely, inner BRB integrity [23], immune regulation [24], autophagy [25], and glucose metabolism [26], are compromised with aging.

It is well established that elevated IOP in mice leads to RGC dysfunction and ultimately degeneration [27-30]. Several but not all studies also report that other retinal neurons such as amacrine cells are negatively impacted by elevated IOP [28, 31-34]. However, the impact of aging in animals with glaucoma remains poorly understood. It has been reported that aging affects the ability of OFF RGCs to adapt and recover when exposed to acute IOP elevations [35], and epigenetic reprogramming of aged, glaucomatous eyes restores RGC resilience, resembling RGCs in younger mice [36]. Our recent works have repeatedly emphasized the effect of OHT on the retinal microvasculature. We reported that in transient, high IOP young mouse models, vascular loss is associated with RGC dysfunction [37], and that chronic, mild ocular hypertension (OHT) results in retinal capillary loss before RGC loss [38]. However, this latter phenotype varies among the three retinal capillary plexi (RCP), depending on the magnitude and chronicity of OHT. Clinical studies with OCT Angiography in glaucoma patients also highlight age as a vascular risk factor [39, 40]. Thus, deleterious age-related changes to retinal capillaries, the BRB, and neurons may serve as a universal trigger and facilitate progression in age-dependent neurodegenerative diseases, like glaucoma. In this manuscript, we aim to understand the distinct and overlapping effects of aging and IOP elevation on retinal neurons and microvasculature.

## METHODS

### Animal use

This study uses exclusively C57BL6J mice from Jackson Laboratory (Cat# 000664). All mouse experiments were conducted as per the guidelines approved by the Institutional Animal Care and Use Committee of Baylor College of Medicine, the ARVO statement for the use of animals in ophthalmic and vision research, and the NIH guide for the use of laboratory animals. Animals in our young data set consisted of a combination of reanalyzed mice [38] and additional mice generated in this study. For all experiments, both sexes were used in approximately equal numbers.

### Induction of ocular hypertension

**Figure 1.**
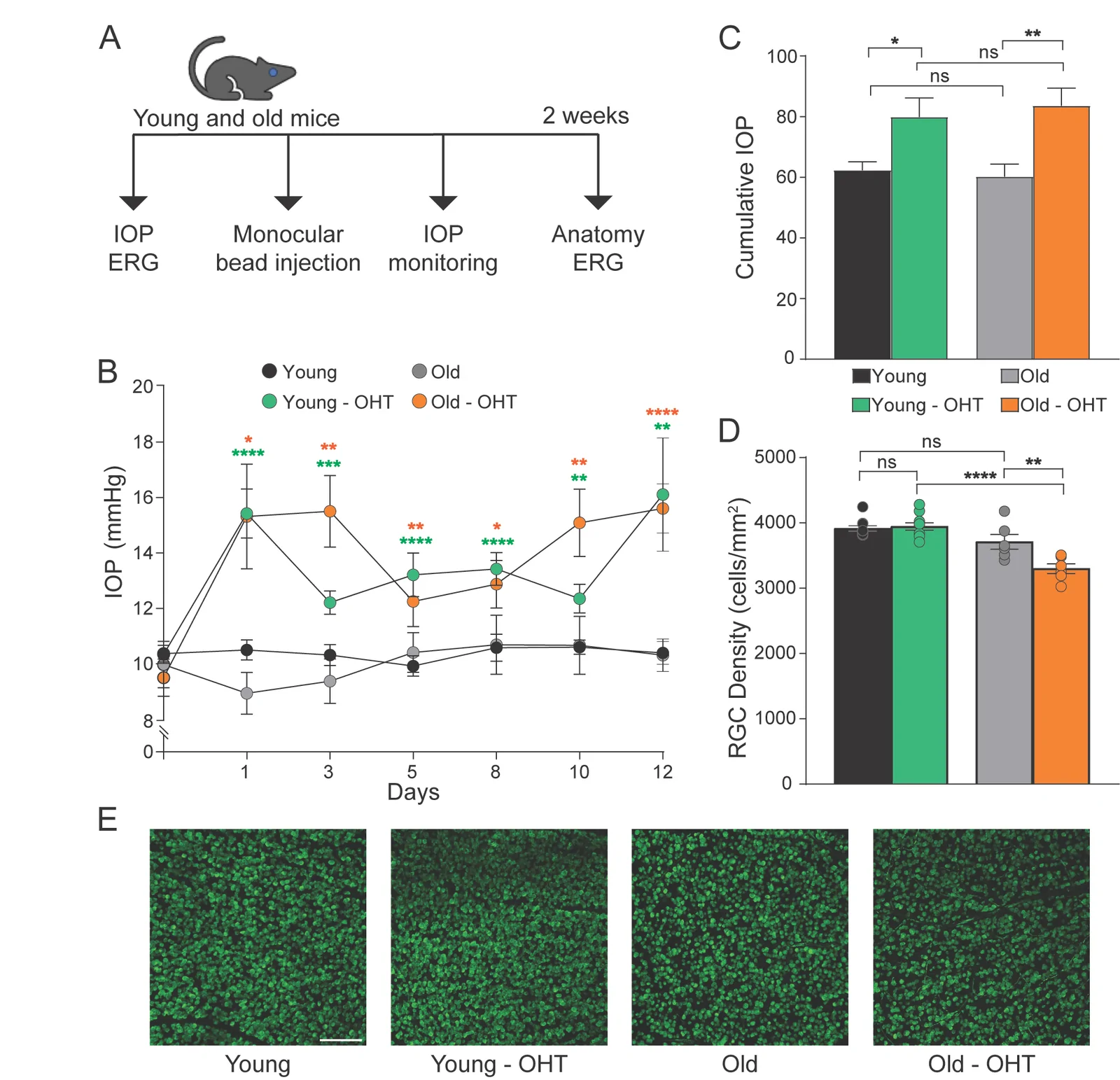
**Induction of ocular hypertension (OHT) and changes in RGC density with age and OHT. (A)** Experimental timeline. Baseline intraocular pressure (IOP) and electrophysiological parameters were recorded for young and old mice. After baseline testing, microbeads were injected into one eye to induce ocular hypertension (OHT). The contralateral eye served as an intra-animal control. At the end of the 2 weeks, anatomical and electrophysiological data were collected and analyzed for both groups. **(B)** Three times a week IOP measurements were taken throughout the experiment for young and old mice [Young and Young - OHT (n=19), Old and Old - OHT (n=8)]. IOP readings were higher in the bead injected eyes for both groups compared to their respective contralateral eyes at all time points (*P<0.05, **P<0.01, ***P<0.001, ****P<0.0001; multiple paired t tests). **(C)** Cumulative IOP calculated by area under the curve (AUC) for the IOP readings from B. Both Young - OHT and Old - OHT eyes had increased IOP compared to contralateral controls (*P<0.05 and **P<0.01, respectively; unpaired t tests). The magnitude of IOP elevation between Young - OHT and Old - OHT eyes was equivalent. **(D)** RGC density was unaffected by aging. RGC density in Young - OHT eyes (n=10) was also unaffected, while in Old - OHT eyes a significant reduction in RGC density was detected (n=6, **P<0.01; one-way ANOVA). **(E)** Representative images of RBPMS immunostained retinas for Young, Young - OHT, Old, and Old - OHT eyes (scale bar = 200 µm).

Ocular hypertension (OHT) was induced in one eye as previously described [28, 38]. Briefly, mice were anesthetized by intraperitoneal injection of a combination anesthetic solution (ketamine 37.5 mg/ml, xylazine 1.9 mg/ml, and acepromazine 0.37 mg/mL). The pupil was dilated with 1% tropicamide and 2.5% phenylephrine while the cornea was anesthetized with 0.5% proparacaine hydrochloride drops. A mix of polystyrene beads (6μm and 1μm diameter polystyrene beads, cat#15715-5, cat#15713-15; Polysciences, Inc., Warrington, PA) were injected into the anterior chamber with a 30g needle (1.75 µL final volume in 12-week mice; 2.5 μl volume in 18-month mice). This injection was followed by 3μL of sodium hyaluronate (cat#571182 Provisc; Alcon Laboratories, Ft. Worth, TX). In aged mice, a greater volume of microbeads was required to achieve sustained IOP elevation equivalent to that of younger mice. This is likely due to the increased size of the anterior chamber in older animals, which would require an increased volume of microbeads to adequately block aqueous outflow [41]. In all cases, the non-injected eye served as a control. Under isoflurane anesthesia, IOP measurements were taken 3 times weekly, using a tonometer (Tonolab), to confirm the induction and maintenance of OHT (Fig. 1A). A total of 38 animals were injected (26 young; 12 old). 11 animals were excluded due to insufficient IOP elevation (7 young; 4 old).

### Immunohistochemistry, image processing, and analysis

Mice were euthanized via cervical disarticulation under isoflurane-induced unconsciousness, followed by decapitation. Retinas were dissected according to previously established protocols [38]. Afterward, the dissected retinal flat-mounts were fixed with 4% paraformaldehyde for 1 h at room temperature followed by 10% donkey serum diluted in phosphate buffered saline with Triton X-100 (PBS++) blocking overnight. Retinas were then incubated with primary antibodies [Collagen IV (EMD Millipore cat#AB756p; 1:300), CD31 (BD bioscience cat#550274; 1:50), and RBPMS (Sigma Aldrich cat#ABN1376; 1:250)] which were diluted with 3% donkey serum in PBS++ buffer (for 5 days at 4°C, followed by overnight incubation at 4°C in secondary antibodies [Alexa fluor 647 donkey anti-rabbit (Jackson Immuno Research Labs cat# 711-605-152; 1:300), Cy3 donkey anti-rat (Jackson Immuno Research Labs cat#712-165-153; 1:300), Alexa fluor 488 donkey anti-guinea pig (Jackson Immuno Research Labs cat#706-545-148; 1:300), and Hoechst 33342 nuclear staining (Invitrogen cat#H3570; 1:1,000)] diluted with 3% donkey serum. For Image analysis, z-stack images of the flat-mount retinas were acquired with laser confocal microscopy (Zeiss LSM 800). 10x images of the entire retina were collected for CD31 staining. 20x images were collected for COLIV, CD31, RBPMS, and Hoechst staining as described previously[38]. RBPMS positive RGCs were manually counted using the ImageJ cell counter plugin. Sholl analysis was performed on 10x CD31 immunostained images to determine complexity of the entire retinal vasculature. Vascular complexity was quantified by calculating the intersection density according to the previously described formula [38] where D_*ring*_ = *intersection density of a full ring; I_obs_= observed number of intersections; r= radius; A_ring_= area of the ring; and δr = radial thickness (1 μm)*.

$$D_{ring}=\frac{I_{obs}}{A_{ring}}=\frac{I_{obs}}{2\pi r\delta r}$$

20x magnification images were separated as stacked images for each retinal plexus for COLIV and CD31 using ImageJ. These images were then analyzed with NIH open-source AngioTool software [42] using a custom workflow[38]. Briefly, this software computes topographical features that define vascular phenotypes namely, junction density (JD - branch points that join the vessel segments), total vessel length (TVL - vessel segments connected by the branch points), vessel area (VA - total vessel segment coverage in the explant of retina), and vessel diameter (VD - relative parameter to determine the vessel width calculated by equation:

$$VD=\frac{VA}{TVL}$$

AngioTool software provided semi-automated quantification which limits investigator bias while identifying vascular phenotypes [42]. Lastly, to measure the capillary loss in the RCPs we measured acellular capillary density [38]. Acellular capillaries were manually counted as the capillaries that are stained with COLIV but not CD31. Next, the total count of these capillaries in the explant was normalized to the vascular area in the COLIV immunostained retinas to determine the acellular capillary density.

### Transmission electron microscopy (TEM)

Dissected eye cups were fixed with 3% glutaraldehyde and then washed in 1M sodium phosphate buffer (pH 7.3). After the wash, eye cups were post-fixed in 1% osmium tetroxide and dehydrated through a series of graded alcohols. The eye cup samples were infiltrated (hardened) with acetone and polybed 812 plastic resin. Following this, samples were embedded in plastic molds with 100% polybed 812 plastic resin. 1µm sections (thick sections) were cut on a Leica EMCU7 ultramicrotome, placed on glass slides, and stained with Toluidine Blue. Second, ultra-thin sections (0.08µm) were cut and mounted on 100 mesh copper grids. These grids were stained with 2% uranyl acetate and Reynold’s lead stain. Using the JEOL JEM 1230 electron microscope, images were captured on an AMTV600 digital camera. 5000x magnification images were acquired to identify and visualize the retinal capillaries while tight junctions and basement membranes were visualized at 12000x magnification.

### Electroretinogram (ERG)

Mice were prepared for ERG recordings according to previously established protocols [28]. Briefly, mice were dark adapted overnight, and all procedures were performed under dim red light. Mice were anesthetized and kept on a heating pad at approximately 35^o^C. Mouse pupils were dilated using 1.0% tropicamide and 2.5% phenylephrine, and cornea anesthesia was achieved using 0.5% proparacaine hydrochloride. Platinum recording electrodes were placed in contact with the center of the cornea and maintained in place with a small amount of 2.5% methylcellulose gel. ERG signals were amplified and bandpass filtered from 0.1 - 1kHz (Grass Instruments, West Warwick, RI). All data were digitized and sampled at a rate of 10 kHz using a data acquisition system (USB-6216, National Instruments, TZ) at a sampling rate of 10kHz. Traces were analyzed using custom software in MATLAB (MathWorks, Natick, MA). Scotopic measurements were generated using cyan-light-emitting diodes of 500 nm peak wavelength. Positive scotopic threshold responses were generated at light intensity ranges from (-)2.29 to (-)1.04 log R*/rod (photoisomerization per rod). A-wave and B-wave were measured at light intensities from 0.20 to 2.87 log R*/rod.

### Statistical analysis

Data throughout are presented as mean ± SEM. Area under the curve (AUC) was calculated for daily average IOP readings and Sholl analysis. To compare the impact of aging in old eyes, the data of vascular phenotypes, acellular capillaries, and ERGs from each old retina were normalized to the average data from young retinas as follows:

$$\left[\frac{Old}{Young\text{ (}average\text{)}}*100\right]$$

To compare the magnitude of the IOP effect in OHT eyes, the data of vascular phenotypes, acellular capillaries, and ERG from each OHT retina were normalized to their respective contralateral retina as follows:

$$\left[\frac{OHT\;eye}{Contralateral\;eye}*100\right]$$

IOP, RGC density, and vasculature datasets were tested for normality using the Shapiro Wilk test. The data sets that were normally distributed were compared using one-way ANOVAs and the appropriate t-tests. Some datasets were not normally distributed and were instead analyzed by Mann-Whitney and Wilcoxon tests (see Figure Legends). ERG measurements were analyzed with repeated measures ANOVA to compare among groups. All statistical analyses were conducted using Prism (GraphPad, La Jolla, CA). The thresholds for statistical significance are represented as *P < 0.05; **P < 0.01; ***P < 0.001; ****P < 0.0001; ns = non-significant.

## RESULTS

### Mild OHT causes RGC loss in old but not young mice

We studied the impact of 2 weeks of mild OHT on retinal anatomy in young and old mice. Injected eyes showed increased IOP compared to their contralateral controls in both young (*P<0.05) and old (*P<0.01) mice (Fig. 1B). The magnitude of OHT was equivalent between age groups (ns; young = 3.07 ± 1.986 mmHg; old = 3.64 ± 0.931 mmHg; Fig. 1C). Baseline RGC density (cells/mm^2^) was not affected by aging (Fig. 1D). As expected, IOP elevation to this level in young OHT eyes did not cause RGC loss (Fig. 1D). In contrast, old OHT eyes showed a RGC loss of 16% (Fig. 1D; **P<0.01). These data suggest that increased age causes increased susceptibility to IOP-induced RGC loss.

### Aging and OHT disrupt retinal vascular complexity

**Figure 2.**
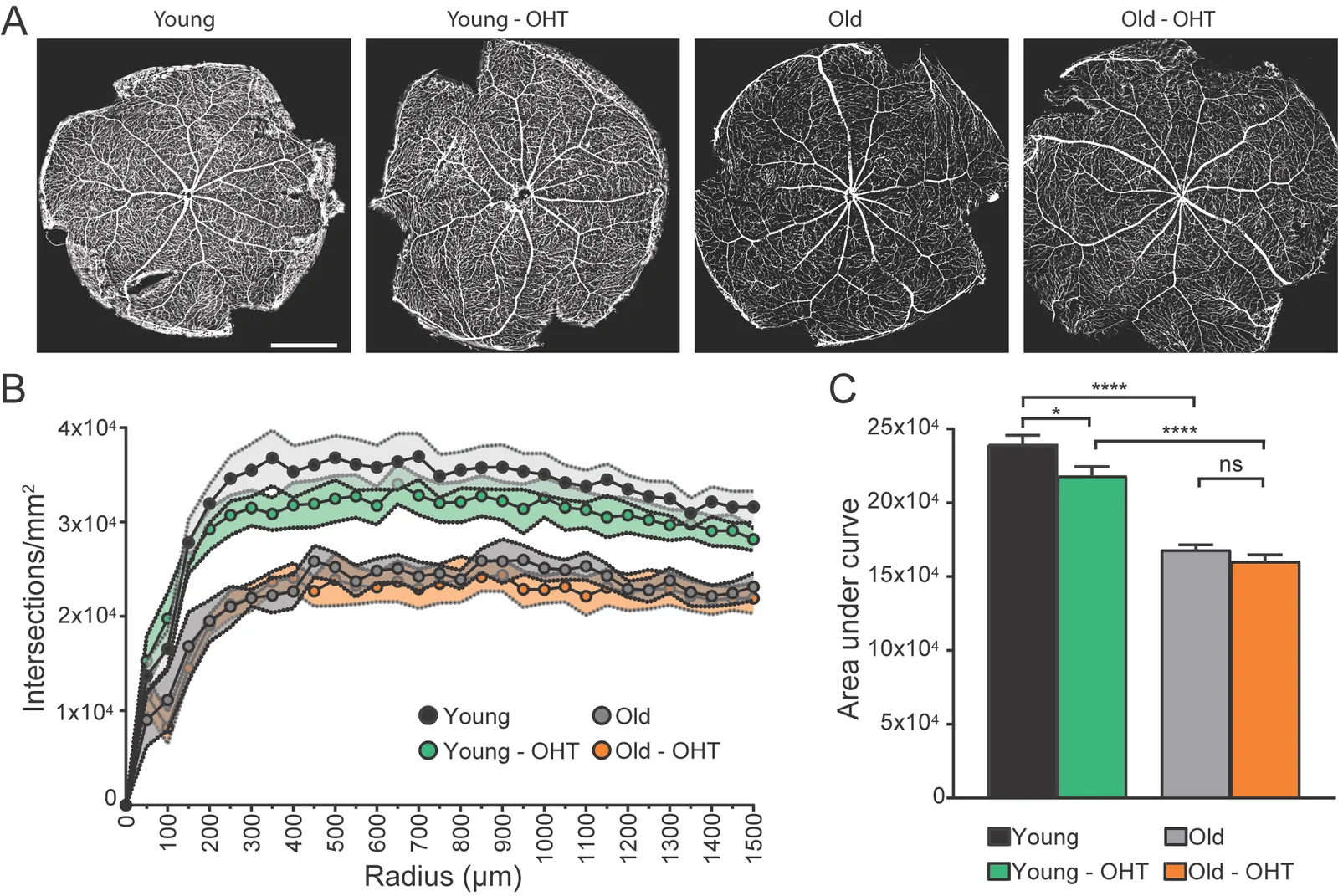
**Reduced retinal complexity with age and OHT. (A)** Representative images of CD31 immunostained retinas for Young, Young - OHT, Old, and Old - OHT eyes (scale bar = 500µm). (**B)** Sholl analysis of vascular complexity for each of the four conditions (Young, Young - OHT [n=10], Old and Old - OHT [n=6]). **(C)** Vascular complexity in Old and Old - OHT eyes was significantly reduced (AUC; ****P<0.0001) when compared with Young and Young - OHT eyes, respectively (one-way ANOVA). Vascular complexity was also significantly lower in Young - OHT eyes compared with contralateral Young eyes (AUC; *P<0.05; one-way ANOVA) but not in Old - OHT eyes compared with contralateral Old eyes.

Next, we studied the anatomy of the retinal microvasculature using CD31, a marker for endothelial cells. We measured retinal complexity by calculating the number of vascular intersections using a Sholl analysis (Fig. 2A). In all retinas, the intersection density gradually increased with distance from the optic nerve, peaking in the mid-periphery, and plateauing in the periphery (Fig. 2B). Old retinas showed significantly reduced retinal complexity compared to young retina controls (Fig. 2C; AUC ****P<0.0001). Young - OHT eyes also displayed reduced vascular complexity compared to contralateral controls (Fig. 2C; AUC *P<0.05). OHT did not cause additional vasculature deficits in old retinas compared to contralateral controls. In summary, we confirmed that OHT in young mice is associated with reduced capillary complexity [38]. Interestingly, aging caused a large reduction in capillary complexity, but this was not further exacerbated by OHT.

**Table 1:** Magnitude of vascular injury in retinal plexi.

Old vs Young	(Old/Young [average]) *100		
	SRCP	IRCP	DRCP
**Junction Density**	88%	79%	91%
**Total Vessel Length**	93%	91%	95%
**Vessel Area**	119%	99%	109%
**Vessel Diameter**	128%	109%	115%
**Acellular Capillary Density**	86%	150%	15%
**Young - OHT vs Young**	(Young - OHT/Young) *100		
	SRCP	IRCP	DRCP
**Junction Density**	102%	81%	94%
**Total Vessel Length**	100%	91%	97%
**Vessel Area**	97%	84%	98%
**Vessel Diameter**	98%	93%	101%
**Acellular Capillary Density**	109%	201%	96%
**Old - OHT vs Old**	(Old - OHT/Old) *100		
	SRCP	IRCP	DRCP
**Junction Density**	90%	83%	88%
**Total Vessel Length**	96%	92%	95%
**Vessel Area**	95%	83%	81%
**Vessel Diameter**	100%	90%	85%
**Acellular Capillary Density**	161%	157%	171%

The magnitude of vascular phenotype changes in Old retinas is expressed as a percent change relative to Young retinas. The magnitude of vascular phenotype changes in Young – OHT and Old - OHT eyes is expressed as a percent change relative to its non-injected, contralateral eyes.

### Aging amplifies OHT-induced changes to retinal capillary plexi

**Figure 3.**
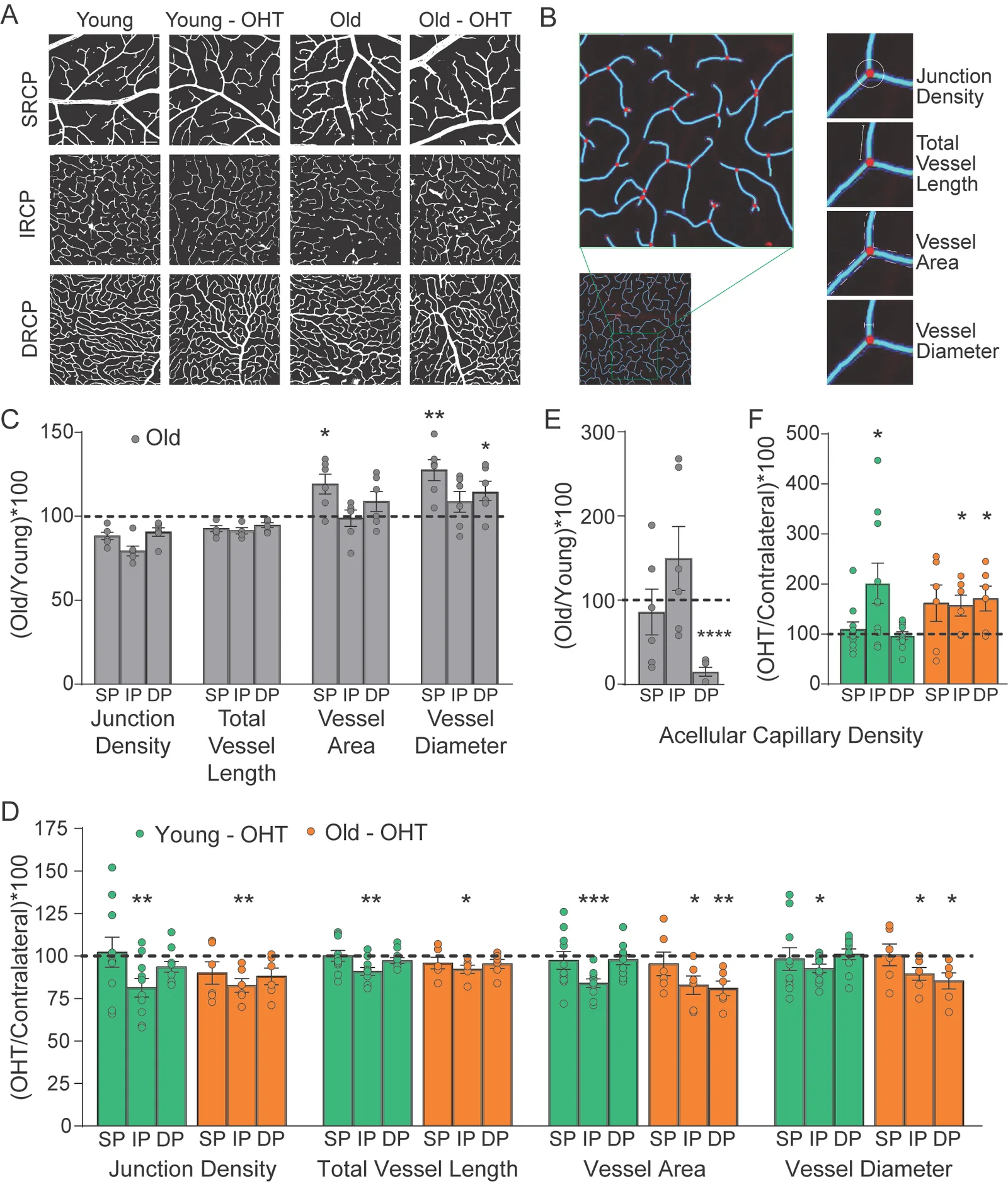
**Diverse vascular phenotypes with age and OHT. (A)** Representative images of a CD31 immunostained superficial retinal capillary plexus (SRCP), intermediate retinal capillary plexus (IRCP), and deep retinal capillary plexus (DRCP). Scale bar = 200 µm. **(B)** Schematic of vascular parameters derived from topographical analysis using AngioTool. Red dots represent junction density (JD), blue segments indicate total vessel length (TVL), cumulative blue and red coverage represents vessel area (VA), and the vessel diameter (VD) is calculated as TVL normalized to VA. **(C)**Vascular phenotypes of Old retinas are expressed as percent change relative to Young retinas. In Old retinas, the SRCP has significantly increased VA (*P<0.05; unpaired t test) and VD (**P<0.01), while the DRCP shows increased VD (*P<0.05).**(D)** Vascular phenotypes of Young - OHT (green) and Old - OHT (orange) retinas are expressed as percent change relative to contralateral controls. In Young - OHT retinas, the IRCP showed significant decreases in JD, TVL, VA, and VD (**P<0.01, **P<0.01, ***P<0.001, and *P<0.05 respectively; paired t tests). In Old - OHT retinas, the IRCP exhibited similar reductions in JD, TVL, VA, and VD (**P<0.01, *P<0.05, *P<0.05, and *P<0.05 respectively; paired t tests), and the DRCP showed significant reduction in VA and VD (**P<0.01 and *P<0.05 respectively; paired t tests). **(E)**Acellular capillary density (ACD) in old retinas is expressed as percent change relative to young retinas. ACD was significantly lower in the DRCP of Old retinas compared to Young retinas (****P<0.0001; unpaired t tests).**(F)** ACD following OHT is shown as percent change relative to contralateral controls. In Young - OHT retinas, ACD was significantly increased in the IRCP (*P<0.05, Wilcoxon test). In Old - OHT retinas, ACD was significantly increased in both the IRCP and DRCP (*P<0.05 respectively; Wilcoxon tests). In all cases (C-F), dotted line = 100%.

Next, we analyzed vascular topography by examining each retinal capillary plexus individually. The three retinal capillary plexi were separated into z-stack images and analyzed using AngioTool (NIH open-source software; Fig. 3A) [38]. We measured four variables: capillary junction density, total vessel length, vessel area, and vessel diameter (Fig. 3B). Raw comparisons are presented in Supplemental Figure 1.

We began by comparing Old to Young retinas across all plexi (Fig. 3C, Table 1). In the SRCP, Old retinas exhibited a 19% increase in vessel area and a 28% increase in vessel diameter (*P <0.05; **P < 0.01 respectively). In the DRCP, junction density was reduced by 9% (*P <0.05); while vessel diameter increased by 15% (*P < 0.05). The IRCP was not significantly impacted by age.

Acellular capillaries, which form when endothelial cells degenerate and leave their basement membrane behind, were quantified as a marker for microvascular degeneration. In Old retinas, we found that acellular capillary density in the DRCP was significantly reduced by 85% (****P<0.0001; Fig. 3E and Table 1) comparedtoYoung retinas. Overall, these results demonstrate that aging significantly alters vascular topography across the retina.

To measure the impact of OHT on vascular phenotypes, we compared Young - OHT and Old - OHT retinas to their contralateral controls. In Young retinas, OHT primarily affected the IRCP, resulting in significant reductions in junction density (19%, **P<0.01), total vessel length (9%, **P<0.01), vessel area (16%, ***P<0.001), and vessel diameter (7%, *P<0.05) (Fig. 3D and Table 1).

In contrast, Old - OHT retinas showed more widespread vascular damage. Similar to Young - OHT eyes, the IRCP showed significant reductions in junction density (17%, **P<0.01), total vessel length (8%, *P<0.05), vessel area (17%, *P<0.05), and vessel diameter (10%, *P<0.05). Additionally, there were changes to the DRCP, with significant decreases in vessel area (19%, **P<0.01) and vessel diameter (15%, *P<0.05) (Fig. 3D and Table 1). There were no significant changes to the IRCP in Old - OHT retinas.

Next, we measured the impact of OHT on vascular pathology by quantifying acellular capillary density. In Young - OHT retinas, acellular capillary density increased in the IRCP by 101% (*P<0.05) while remaining largely unchanged in the SRCP and DRCP (Fig. 3F and Table 1). In contrast, Old - OHT retinas showed significantly increased acellular capillary density in both the IRCP and DRCP by 57% (*P<0.05) and 71% (*P<0.05) respectively (Fig. 3F and Table 1). Taken together, these findings suggest that OHT primarily disrupts the IRCP in young retinas, whereas in old retinas, vascular disruption is generalized across multiple plexi.

### Aging and OHT disrupt blood-retina-barrier (BRB) anatomy

**Figure 4.**
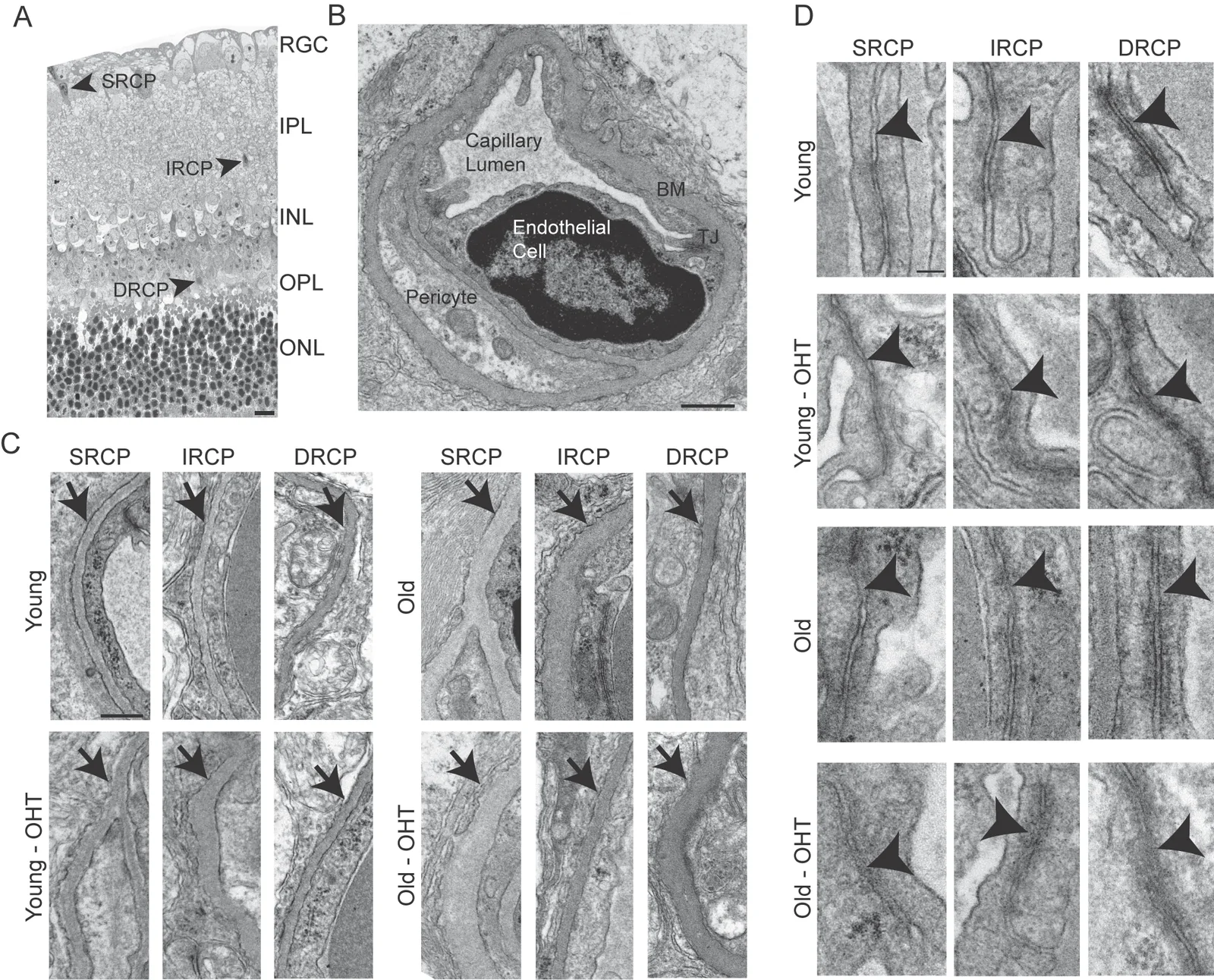
**TEM changes with age and OHT. (A)** Transmission electron microscopy (TEM) image of a retinal cross section (scale bar = 10 µm). **(B)** TEM image of a normal IRCP showing an endothelial cell, a pericyte, basement membrane (BM) and tight junction (TJ). Scale bar = 800 nm. **(C)** Representative TEM images of BM in Young, Young - OHT, Old, and Old - OHT eyes (arrows). Basement membrane thickness (BMT) was increased in all three RCPs in Old eyes. BMT is also increased in the IRCP of Young - OHT eyes and decreased in the IRCP of the Old - OHT eyes. BMT was also increased in the SRCP and DRCP of Old – OHT eyes. Scale bar = 800 nm. **(D)** TEM images of TJs in Young, Young - OHT, Old, and Old - OHT eyes. Young eyes displayed normal, intact TJs (arrowheads). The continuity of the TJs was affected in Old eyes, showing focal areas of disruption (arrowheads). In OHT eyes, Young - OHT retinas showed a combination of focal and generalized disruption and Old - OHT retinas showed ubiquitously disrupted TJs across all RCPs (arrowheads). Scale bar = 200 nm. Qualitative assessments for each condition were performed on three replicates of each plexus.

The varied vascular changes we observed suggested a possible disruption of the BRB [43-46]. Therefore, we used TEM to analyze basement membrane and tight junction topography in Young, Old, and OHT retinas. First, we identified retinal plexi according to anatomic location in retinal sections (Fig. 4A and 4B). Qualitative observation showed that basement membrane thickening in all three plexi in the Old retinas compared to Young retinas. OHT also impacted basement membrane anatomy, but in a variable way. In Young - OHT retinas, there was basement membrane thickening, primarily in the IRCP, with subtle differences observed in the SRCP and DRCP. Conversely, in Old - OHT eyes, the basement membrane was thinned in the IRCP and thickened in the SRCP and DRCP compared to contralateral controls (Fig. 4C).

**Figure 5.**
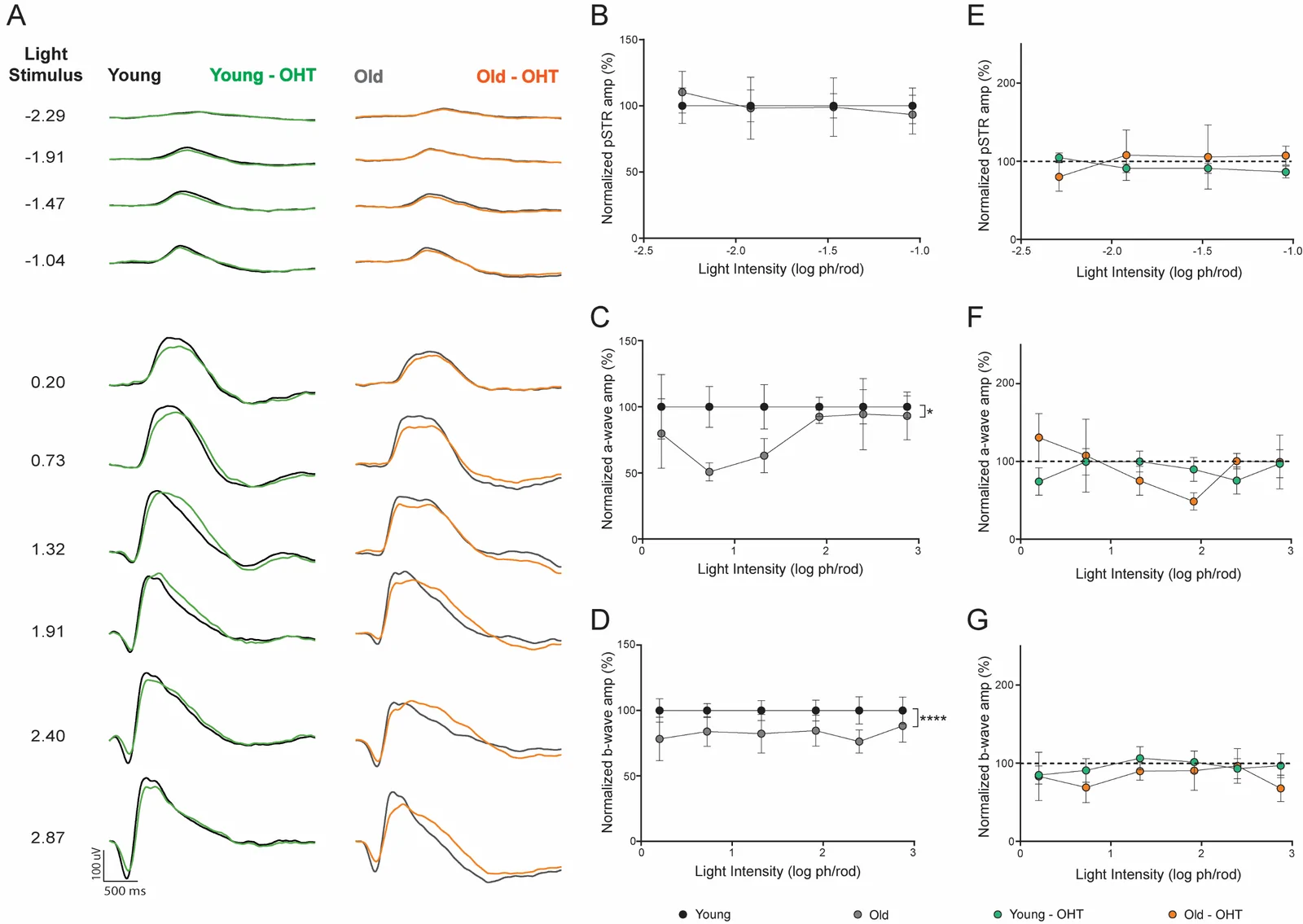
**Effects of age and OHT on the electroretinogram. (A)** Averaged electroretinogram (ERG) waveforms of young and old eyes are displayed across a range of light intensities (-2.29 to 6.17 log photoisomerization per rod). In each case, OHT waveforms are overlaid with contralateral control waveforms within their respective age cohorts. **(B-D)** Peak ERG amplitudes of Old eyes were compared to Young eyes as a percentage across a range of light intensities. While no significant difference is observed in the pSTR amplitudes of Old eyes **(B)**, there is a significant reduction in both A-wave (C,*P<0.05; repeated measures ANOVA) and B-wave (D,****P<0.0001; repeated measures ANOVA) amplitudes in Old eyes compared to Young eyes. **(E-G)** The effects of OHT were investigated by normalizing the amplitudes of OHT eyes to their contralateral eyes. Elevated IOP did not significantly affect the pSTR **(E)**, A-wave **(F)** or B-wave **(G)** amplitudes in Old - OHT eyes or Young - OHT eyes compared to their contralateral eyes [Young, Young - OHT (n=8); Old, Old - OHT (n=5)].

Tight junction anatomy in young retinas was normal, as expected (Fig. 4D). However, in old retinas, we frequently observed small, focal areas where tight junction integrity was compromised in all three plexi. In Young - OHT retinas, we observed both segmented and generalized disruption of tight junctions across all three plexi. Old - OHT eyes exhibited the most severe tight junction phenotype, with widespread disruption across all three plexi. These findings suggest that both aging and OHT contribute to tight junction breakdown, with the combined effects being most pronounced in old retinas exposed to OHT.

### Aging disrupts normal retinal electrical function but does not exacerbate OHT-induced dysfunction

Using the electroretinogram (ERG), we recorded electrophysiological responses of the retina across a range of light stimulus intensities that cover approximately 4 log units (Fig. 5A). We compared the retinal function in old eyes by normalizing ERG waveform peak amplitudes to those of young control eyes. Across all age groups, we observed a characteristic increase in ERG amplitudes in response to increasing light intensities. Old mice showed normal RGC function, as indicated by pSTR amplitudes, compared to young controls (Fig. 5B). However, old mice exhibited significantly reduced a-wave (Fig. 5C; *P < 0.05) and b-wave amplitudes (Fig 5D; ****P < 0.0001) compared to young controls, reflecting diminished photoreceptor and bipolar cell activity, respectively. We next investigated the effect of OHT on retinal function by normalizing ERG amplitudes in OHT eyes to their contralateral control eyes. In both age groups, we found that retinal function was generally preserved after two weeks of OHT (Fig. 5E-G).

## DISCUSSION

Previously, we demonstrated that the IRCP exhibits selective vulnerability to OHT on a time scale that precedes RGC loss. Our current results build upon and expand those findings by showing that aging exacerbates this vulnerability. We confirmed that OHT in young retinas primarily disrupted the IRCP, with reductions in capillary junction density, vessel length, and vessel area. These changes were accompanied by a marked increase in acellular capillaries. Similarly, the IRCP displayed basement membrane thickening and localized tight junction disruption, reinforcing its central role in early glaucomatous changes. In old mice, we found that OHT damage was more widespread and variable across all three retinal plexi. Indeed, the general increase in acellular capillaries and significant structural disruptions in old OHT eyes show that aging reduces the resilience of all vascular layers. However, the IRCP still exhibited pronounced and distinct deficits, aligning with its established vulnerability in the early stages of glaucomatous damage.

Aging is the most important non-modifiable risk factor for glaucoma, suggesting that age-related alterations to RGCs or their supporting tissues heighten vulnerability to damage [47, 48]. Directly, aging is associated with increased mitochondrial dysfunction, oxidative stress, and reduced autophagy in RGCs[49-53]. Indirectly, aging decreases blood flow, disrupts the BRB, and contributes to capillary stiffness [54]. All these mechanisms have been shown to be worsened in the context of glaucoma [55-57].

Our vascular phenotypes in Old, Young - OHT, and Old - OHT retinas largely align with known patterns of microvascular damage associated with both aging and glaucoma [58-60]. Notably, for most vascular features, the effects of aging are exacerbated by the presence of OHT. However, vessel area and diameter show an interesting divergence: aging increases these metrics while OHT decreases them. In aging, altered metabolic demand may lead to reduced ocular blood flow while the accompanying decline in vessel density could trigger compensatory enlargement of remaining vessels to maintain adequate perfusion [60, 61]. At the cellular and molecular levels, this increase in vessel area and diameter in aging may be linked to endothelial cell and pericyte dysfunction, increased vascular stiffness, oxidative stress, and cellular senescence [59, 60, 62]. In contrast, OHT induces a narrowing of vessel area and diameter, consistent with prior reports implicating endothelial cell damage, inflammatory responses, and impaired neurovascular coupling [14, 60, 63-65]. The overlapping yet distinct vascular responses to aging and OHT suggest that while both conditions compromise vascular integrity, they likely do so through different mechanisms.

To further support the idea that OHT and aging drive vascular dysfunction through differing mechanisms, we found that the IRCP was significantly altered by OHT in both young and old retinas, while it was unaffected by aging alone. Previously, we have shown that OHT induces hypoxic stress, and that the IRCP is particularly sensitive to damage under these conditions [38]. This hypoxia may be a key driver underlying the disparate vascular outcomes observed through OHT. Together, these findings suggest a distinct OHT-specific mechanism of vascular dysfunction that operates independently of aging.

This and previous studies highlight the temporal sequence of experimental OHT in which vascular compromise precedes RGC loss [38]. Our results build upon this insight by demonstrating that aging accelerates the impact of OHT on RGC survival. While young OHT retinas showed no significant RGC loss after two weeks of mild IOP elevation, old OHT retinas exhibited moderate RGC loss after the same duration of equivalent IOP elevation. One potential explanation for this finding is that the aging retina’s diminished vascular integrity may accelerate hypoxic and metabolic stress, driving earlier and more extensive RGC loss. Along these lines, we found that aging contributed to basement membrane thickening and localized tight junction disruptions, particularly in the IRCP, and OHT amplified these effects. The combination of aging and OHT resulted in severe and widespread tight junction instability and basement membrane alterations. Since tight junction disruption is likely to contribute to capillary permeability, this anatomic site may be the physical link between vascular dysfunction and subsequent neurodegeneration [66, 67].

Using the ERG, we found that aging decreased retinal function, but this dysfunction was not further exacerbated with OHT. These age-related effects are consistent with prior studies [35, 68]. One possible explanation is that the aged retina may increase its intrinsic excitability to compensate for IOP-related dysfunction [35, 69]. A key limitation of the ERG is that it measures global electrical activity, which may not capture subtle or localized changes related to synaptic remodeling, metabolic shifts, or other intrinsic compensatory mechanisms. Additionally, it is possible that retinal dysfunction reaches a ceiling effect, where age-related decline masks or occludes further impairments induced by OHT. This study was limited to two timepoints (12 weeks, 18 months). It is unclear whether age-related dysfunction occurs gradually or accelerates rapidly with advancing age. Future studies will investigate intermediate time points to further elucidate the speed and pattern of age-related deterioration.

In conclusion, we found that aging causes multiple kinds of retinal vascular remodeling. These changes modify and exacerbate the effects of OHT ultimately leading to RGC loss. This study highlights the importance of age in experimental studies of glaucoma in mice and argues for careful comparisons between mice of similar ages going forward.

## Supplementary Materials

The Supplementary data can be found online at: www.aginganddisease.org/EN/10.14336/AD.2025.0349.
