# Supplementary material for: Advanced Age Worsens Phenotypes of Ocular Hypertension in Mice
Source: Aging Dis. 2025 May 28;17(3):1664–76. doi: 10.14336/AD.2025.0349 (PMC13061565; doi:10.14336/AD.2025.0349)
Supplement: Supplementary file 1 — The Supplementary data can be found online at: www.aginganddisease.org/EN/10.14336/AD.2025.0349. [file AD-17-3-1664-s.pdf]

SUPPLEMENTARY DATA

Advanced Age Worsens Phenotypes of Ocular Hypertension in Mice

Priyamvada M. Pitale, Solomon E. Gibson, Caroline C. Keehn, Arman T. Yazdian, Guofu Shen, Benjamin J. Frankfort

© 2025. Pitale PM et al. Published online at http://www.aginganddisease.org/EN/10.14336/AD.2025.0349

SUPPLEMENTARY DATA

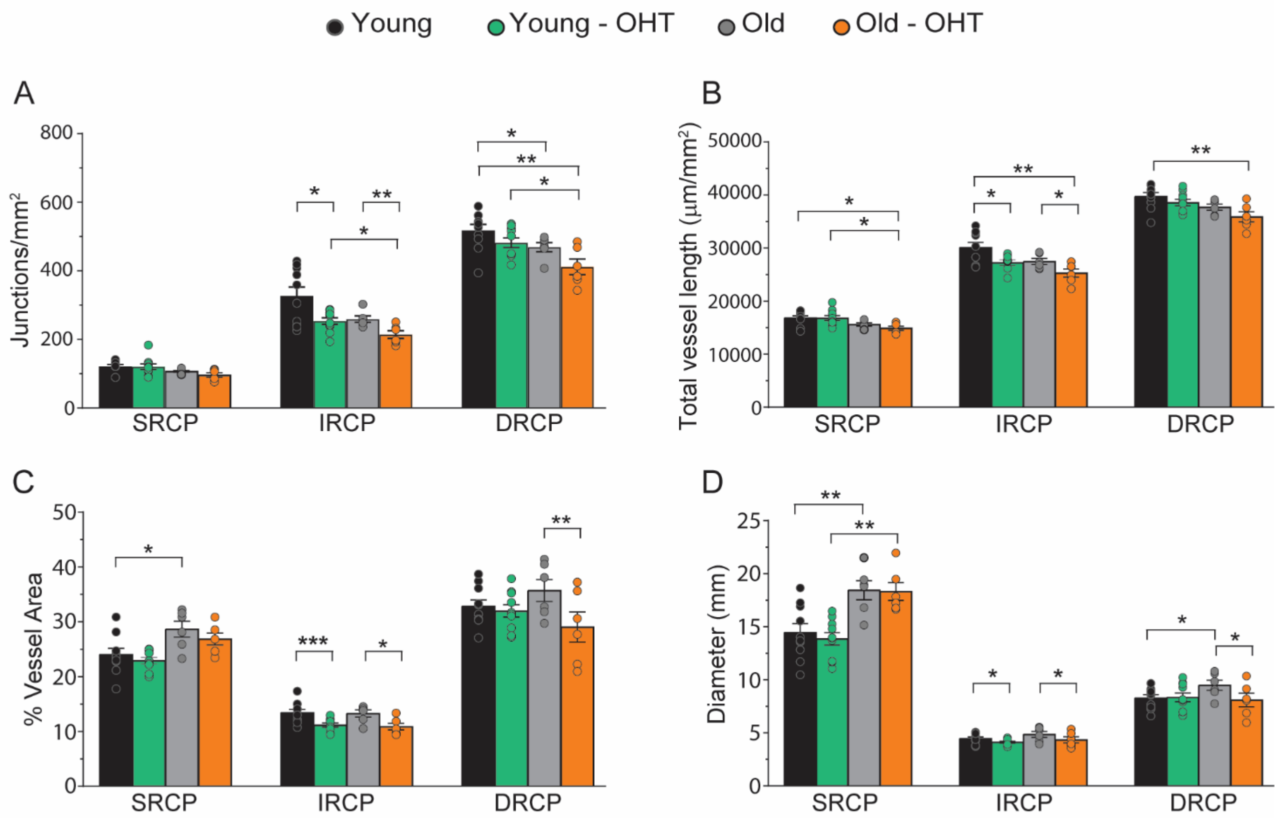

Supplementary Figure 1. Detailed vascular phenotypes for all RCPs. **(A-D).** Extended data for CD31 immunostained retinas. **(A)** Junction Density (JD, number of junctions/mm^2). No significant changes were seen in the SRCP for Young, Young - OHT, Old, and Old - OHT retinas. In the IRCP, a significant reduction in the JD was observed in Young - OHT (*P<0.05; paired t test) and Old - OHT (**P<0.01; paired t test) retinas compared to contralateral retinas. In addition, Old - OHT retinas showed significantly reduced (*P<0.05; unpaired t test) JD compared to Young - OHT retinas. In the DRCP, Old and Old - OHT retinas both had a significant reduction (*P<0.05; Mann Whitney test and **P<0.01; unpaired t test, respectively) in JD compared to Young retinas. Finally, Old - OHT retinas showed significantly reduced (*P<0.05; unpaired t test) JD compared to the young - OHT retinas. **(B)** Total vessel length (TVL, μm/mm^2). In the SRCP, TVL in Old - OHT retinas was reduced compared to Young and Young - OHT retinas (*P<0.05; unpaired t test). Old - OHT retinas showed significantly reduced (*P<0.05; unpaired t test) TVL compared to Young - OHT retinas. In the IRCP, a significant reduction in TVL was observed in Young - OHT and Old - OHT retinas (*P<0.05; paired t tests) compared to contralateral retinas. In addition, Old - OHT retinas showed significantly reduced (**P<0.01; unpaired t test) TVL compared to Young retinas. In the DRCP, Old - OHT retinas had a significant reduction (**P<0.01; unpaired t test in TVL compared to Young retinas. **(C)** % vessel area (VA). In the SRCP, VA increased in Old retinas compared to Young retinas (*P<0.05; unpaired t test). In the IRCP, VA was significantly reduced in Young - OHT (***P<0.001; paired t test) and Old - OHT (*P<0.05; paired t test) retinas compared to contralateral retinas. In addition, in the DRCP, VA was reduced in Old - OHT retinas (**P<0.01; paired t tests) compared to contralateral Old retinas. **(D)** Vessel diameter (VD). In the SRCP, VD was higher in both Old and Old - OHT retinas (**P<0.01; unpaired t test) compared to Young and Young - OHT retinas. In the IRCP, VD was significantly reduced in Young - OHT and Old - OHT (*P<0.05; paired t tests) retinas compared to contralateral retinas. In the DRCP, VD was increased in Old retinas compared to Young retinas (*P<0.05; unpaired t test). Lastly, VD was significantly reduced in Old - OHT retinas (*P<0.05; paired t tests) compared to contralateral Old retinas.

© 2025. Pitale PM et al. Published online at http://www.aginganddisease.org/EN/10.14336/AD.2025.0349
